# Nonresponse to an item assessing firearm ownership: Associations with suicide risk and emotional distress

**DOI:** 10.1111/sltb.13121

**Published:** 2024-08-30

**Authors:** Samantha E. Daruwala, C. Rosie Bauder, Melanie L. Bozzay, Craig J. Bryan

**Affiliations:** ^1^ The Ohio State University Wexner Medical Center Columbus Ohio USA; ^2^ VA Center of Excellence for Suicide Prevention VA Finger Lakes Healthcare System Canandaigua New York USA

**Keywords:** firearms, nonresponse, suicide risk

## Abstract

**Introduction:**

Firearms account for more than half of suicide deaths in the United States (US) and both ownership and access are associated with increased risk of intentional and unintentional injury. Despite evidence linking ownership and access to suicide risk, individuals may be reticent to answer questionnaire items assessing ownership. The current study examined characteristics of individuals who do not provide a response (nonresponders) to a firearm ownership item in a community sample.

**Methods:**

Data were collected from a cross‐sectional online survey of 10,625 US adults. Univariate and multivariate analyses were conducted to examine demographic, emotional distress, and suicide risk differences across three groups (firearm owners, nonowners, and nonresponders).

**Results:**

Nonresponders were significantly younger, more likely to be female and non‐White than firearm owners and nonowners. Nonresponders were less likely to endorse recent suicidal ideation and probable PTSD than firearm owners, but more likely to endorse probable PTSD than nonowners. Firearm owners were significantly more likely to report several correlates of suicide risk than nonowners.

**Conclusions:**

Nonresponders may be a unique subgroup with distinct demographic, emotional distress, and suicide risk profiles compared to both firearm owners and nonowners. Implications of these findings for future directions are discussed.

## INTRODUCTION

The number of firearm homicides and suicide deaths in the United States (US) reached record highs in 2021, with more than half of firearm‐related deaths being suicides (Davis et al., 2023). Firearms are the most lethal method for suicide, with 90% of suicide attempts with a firearm resulting in death (Conner et al., 2019). Firearm access is common in the US; in a recent study, 40% of US adults reported living in a household with a firearm and 30% reported personally owning a firearm (Van Green, 2021). Firearm ownership and access is associated with an increased risk of suicide (Shenassa et al., 2004) for all household members (Miller et al., 2016). An important strategy for reducing the risk of firearm suicide is using secure firearm storage practices (e.g., in a locked location), which can decrease access to a highly lethal mean during a suicidal crisis (Bryan et al., 2011; Simonetti & Brenner, 2020). Indeed, research has found that secure firearm storage practices are associated with a lower risk of suicide (Monuteaux et al., 2019; Shenassa et al., 2004). Given that firearms account for a large proportion of suicide deaths in the US, it is crucial to understand the experiences and perspectives of firearm owners and those residing in firearm‐owning households to inform suicide prevention strategies at the individual and public health level. Unfortunately, such efforts can be hindered by individuals who are unwilling to answer questions about firearm ownership and access. As a result, we may not be capturing the full range of perspectives, which can hinder the development and implementation of interventions that can reduce the risk of firearm suicide (Bryan et al., 2021).

According to findings from the General Social Survey, refusals to answer questions about firearm ownership have increased since 1973, and the rates nearly tripled from around 1% in 2000 to approximately 3% in 2016 (Urbatsch, 2019). Compared to the overall trend in the general population, the rates of refusal to answer may be slightly higher among military personnel and veterans. Studies with military personnel and veterans suggest that only a small percentage of individuals (4%–9%) tend to decline to answer firearm‐related items on a survey (Bryan et al., 2021; Cleveland et al., 2017). The decision to not provide a response to certain survey items may not occur at random, and this subgroup of nonresponders may differ from other participants in important ways (Cook & Ludwig, 1997). Individuals may decline to answer survey questions related to topics that are sensitive in nature or considered taboo, are personally relevant to them, and/or out of concerns that their answers will lead to negative consequences. For example, prior studies have found that individuals with missing data on items assessing suicidal thoughts and behaviors are more likely to report emotional distress and proxy measures of suicide risk (Podlogar et al., 2016; Stanley et al., 2022). Regarding firearms, individuals may perceive they will be stigmatized if they were to disclose owning firearms and be hesitant to answer such items that may identify their firearm ownership status (Kleck et al., 2009). Additionally, individuals can acquire firearms in illicit ways and/or may have unregistered firearms, which may increase concerns about the legal consequences of answering such survey items (Kellermann et al., 1990; Urbatsch, 2019). For instance, qualitative interviews with primary care patients revealed that several participants were apprehensive to disclose their firearm access due to concerns about privacy, autonomy, and their rights to own firearms (Richards, Hohl, et al., 2021).

Extant research has examined the prevalence and variations in responding to survey items about firearm ownership and access. Declining to answer an item assessing firearm ownership on the General Social Survey is more prevalent among individuals who are older and identify as Republicans (Urbatsch, 2019). Blosnich et al. (2021) compared individuals who refused to answer a federal health survey item about firearms in the household with those who provided a valid answer (i.e., selected “yes”, “no”, or “don't know/not sure”) and found that men and White individuals were more likely to refuse to answer the item than women and Latino/a individuals, respectively. Such demographic characteristics are common among firearm owners (Parker et al., 2017), which further suggests that refusing to answer a firearm item may not be missing at random (Blosnich et al., 2021). A recent study by Bond et al. (2023) used several demographic and intrapersonal variables to determine potential underreporting of firearm ownership and identified three unique subgroups of potential under‐reporters. The largest subgroup of potential firearm owners consisted mostly of women, those who identify with another race than Black or White, unmarried, living in urban environments, and do not have kids residing in the home, which differs from the typical demographic characteristics of firearm owners.

To our knowledge, only two studies have examined response variations to a firearm item in relation to suicide risk. Bryan et al. (2021) found that military service members and Veterans who selected “refuse to answer” to an item assessing firearm availability in or around the home were demographically similar to those who endorsed firearm access but endorsed higher rates of probable depression and recent thoughts of death or self‐harm. This finding suggests that individuals who are at higher risk for suicide with access to firearms may be less likely to self‐report their firearm access (Bryan et al., 2021). Using electronic health records, Richards and colleagues (2021) examined patterns among patients who provided a “yes” or “no” response to an item on firearm access versus leaving the item unanswered in a sample of patients who were receiving care for mental health and/or substance use in a primary care or outpatient mental health clinics. Compared to those who provided a “yes” or “no” response, leaving the item unanswered (i.e., nonresponse) was associated with older age, male sex, rural residence, and a substance use disorder diagnosis in the past year. Yet, nonresponse was not associated with suicidal ideation endorsed on the ninth item of the Patient Health Questionnaire‐9 (Kroenke et al., 2001) nor suicide attempt diagnosis (Richards, Kuo, et al., 2021). Findings from these two studies may differ for several reasons, including the populations of interest, the groups being compared within each sample, and how data were obtained.

Overall, prior research suggests that some subgroups of individuals that are likely underreporting firearm ownership may be demographically different from “typical” firearm owners. Further research is needed to further understand the characteristics of individuals who provide a nonresponse to an item assessing firearm ownership and how response variations are associated with suicide risk. To build upon recent findings, the current study examines the demographic, psychiatric, and suicide risk‐related characteristics of individuals who do not provide a response (i.e., nonresponse) to a firearm ownership item in a community sample of adults. In line with prior research (Bryan et al., 2021), we hypothesized that individuals who provided a nonresponse about firearm ownership would be demographically similar to those who endorse firearm ownership and demographically different from those who denied ownership. We also expected that providing a nonresponse to a question about firearm ownership would be positively correlated with emotional distress (i.e., probable depression and probable Post‐Traumatic Stress Disorder [PTSD]) and suicide risk (i.e., recent suicide ideation [SI], past‐month SI, and past‐year SI, recent suicide‐related cognitions). Similar to Bryan et al. (2021), we examined probable depression and probable PTSD as indicators of emotional distress. Prior research suggests that there are inconsistencies in endorsement based on how a self‐report item assessing suicidal ideation is worded (Ammerman et al., 2021). Due to this, we examined multiple methods for assessing suicide risk to determine if variability in how the construct is assessed matters.

## METHODS

### Participants and procedures

Data were collected from March to April 2020 using an anonymous online cross‐sectional survey managed by Qualtrics Panels, an online platform that maintains a database of U.S. residents who are willing to participate in survey‐based research. A quota sampling approach was used to enroll a sample that was demographically similar to 2010 U.S. census demographic distributions. Individuals who were at least 18 years of age were invited to participate in the study via email, which included a hyperlink to the consent form on the survey's landing page. After providing consent to the survey, individuals were directed to the full survey. Participants were not required to provide responses to all survey items; thus, they could skip items if they desired. Upon completion, participants were financially compensated in the amount that was agreed upon when they initially joined the panel. Additional procedures and data quality strategies are outlined by Bryan and colleagues (2022). In total, 10,625 participants completed the survey. Study procedures were reviewed and approved by the institutional review board prior to data collection.

### Measures

#### Demographics

Participants were asked to answer several self‐report items assessing demographics, such as age, gender, race, and prior or current military service status.

#### Firearm ownership

Participants were asked to select one of four response options (“yes”, “no”, “don't know/unsure”, and “refuse to answer”) in response to an item assessing their firearm ownership (i.e., “Do you personally own a gun or firearm?”). For the purposes of the current study and due to low cell counts, participants who selected “don't know/unsure” (*n* = 69) or “refuse to answer” (*n* = 156) were excluded from analyses. Individuals who did not select from the four provided response options (i.e., left the item unanswered) were categorized as “nonresponders”.

#### Probable PTSD

The five‐item Primary Care PTSD Screen for DSM‐5 (PC‐PTSD‐5; Prins et al., 2016) was used to assess PTSD symptoms in the past month. Participants were first asked if they had experienced a traumatic event in their lifetime. Those who reported a trauma history were then asked to use a yes/no response format to answer five items corresponding to the DSM‐5's criteria for PTSD (American Psychiatric Association, 2013). Scores on the PC‐PTSD can range from 0 to 5. A total score of 3 has been identified as the optimally sensitive cutoff point that demonstrated acceptable specificity (>0.80) in a veteran primary sample (Prins et al., 2016). Within a civilian primary care sample, Willamson and colleagues (2022) found that while a cutoff score of 4 had the highest level of sensitivity while maintaining acceptable specificity, a score of 3 still maintained a high level of sensitivity with lower specificity (0.69). Based on recommendations from these findings, the current study used a total score of 3 or higher to define probable PTSD (Prins et al., 2016; Williamson et al., 2022).

#### Probable depression

The nine‐item Patient Health Questionnaire‐9 (PHQ‐9; Kroenke et al., 2001) was used to assess depressive symptoms within the past 2 weeks. Participants reported the frequency of depressive symptoms in the past 2 weeks using a 4‐point scale from 0 (not at all) to 3 (nearly every day). The initial validation study of the PHQ‐9 found that a cutoff score of 10 had appropriate sensitivity (0.88) and specificity (0.88) for major depression (Kroenke et al., 2001). Thus, a total score of 10 or higher was classified as probable depression.

#### Suicidal ideation

Two distinct measures were used to assess suicidal ideation: (1) the ninth item of the PHQ‐9 (Kroenke et al., 2001) and (2) the suicidal ideation item from the self‐report version of the Self‐Injurious Thoughts and Behaviors Interview‐Revised (SITBI‐R; Fox et al., 2020). The ninth item of the PHQ‐9 (Kroenke et al., 2001) asks participants to rate the frequency of “thoughts that you would be better off dead, or of hurting yourself in some way” in the last 2 weeks using a scale from 0 (not at all) to 3 (nearly every day). A nonzero response on the ninth item was used to define recent SI. On the SITBI‐R (Fox et al., 2020), participants were asked if they had ever experienced “thoughts of killing yourself” in their lifetime. Those who endorsed “yes” were then asked to select when they had most recently experienced the thought using three response options (within the past month, within the past year, or more than 1 year ago). Binary variables were constructed using responses to this item to represent past‐month and past‐year presence of suicidal ideation. We chose to use two measures of suicidal ideation due to variations in the wording across both items. The PHQ‐9 is widely used in medical settings like primary care; however, the language used in the ninth item does not reflect recent definitions of suicide ideation that include specific thoughts of engaging in suicide‐related behavior (Bryan & Rudd, 2023; Office of the Surgeon General (US) & National Action Alliance for Suicide Prevention (US), 2012). Due to this, we also used the self‐report SITBI‐R item because the item language aligns with more recent definitions of SI and uses similar wording as other commonly used screening tools (e.g., Columbia Suicide Severity Rating Scale; Posner et al., 2011).

#### Suicide cognitions

The 16‐item Suicide Cognitions Scale‐Revised (SCS‐R; Bryan, May, et al., 2022) was administered to assess cognitive vulnerability to acute suicidal states. Participants used a 5‐point Likert scale (0 “strongly disagree” to 4 “strongly agree”) to rate how their level of agreement with statements reflecting suicide‐specific beliefs and schemas. Higher total scores on the initial SCS and SCS‐R have been shown to be associated with an increased risk for suicidal behaviors, even among those denying suicidal ideation or prior suicide attempts (Bryan et al., 2014; Bryan, May, et al., 2022).

### Data analytic approach

We used a similar data analytic plan as outlined by Bryan et al. (2021). Responses to the firearm ownership item were used to categorize participants into one of three groups: (1) firearm owners (selecting “yes”), (2) non‐firearm owners (selecting “no”), and (3) nonresponders (left the item unanswered). Predictor variables of interest included age, gender (Female vs. Male),^i^ race (Non‐White vs. White), current or prior military service (No vs. Yes), probable PTSD (No vs. Yes), probable depression (No vs. Yes), recent ideation as measured by the PHQ‐9 (No vs. Yes), past‐month SI as measured by the SITBI‐R (No vs. Yes), past‐year SI as measured by the SITBI‐R (No vs. Yes), and recent suicide cognitions (i.e., SCS‐R total). A series of multinomial logistic regression models were used. Univariate models were first used to examine each predictor as an independent correlate of the response group. Multivariate models were then constructed to examine the unique contributions of the emotional distress and suicide risk variables when entered simultaneously. A total of four models were separately constructed for each suicide‐related construct and included the demographic and emotional distress variables that were statistically significant at the univariate level. To aid in interpretation, continuous variables (i.e., age and SCS‐R total) were standardized using z‐scores before being entered as predictors in the analyses.

## RESULTS

A total of 10,400 participants provided a yes (26.2%), no (60.4%), or nonresponse (11.8%) to the firearm ownership item and were included in the subsequent analyses. The subsample primarily consisted of White (62.1%), female‐identifying (50.7%), non‐Hispanic/Latino/a (64.9%) individuals with a mean age of 45.2 years (SD = 16.96). Less than 1% of data was missing for each of the clinical variables [i.e., probable depression (0.6%), probable PTSD (0.2%), recent ideation (0.3%), past‐month SI (0%), past‐year SI (0%), and suicide cognitions (0.1%)].

### Univariate analyses

Table 1 summarizes differences in demographic and clinical variables across the three groups. Of the nonresponders, none reported current or prior military service; due to this, we were unable to include military service in our models. Nonresponders significantly differed on several demographic variables compared to firearm owners and nonowners. Nonresponders (*M* = 34.18) were significantly younger than firearm owners (*M* = 47.42) and nonowners (*M* = 46.44). Nonresponders were less likely to be male (*OR*s = 0.27–0.56) and White (*OR*s = 0.17–0.32) than firearm owners and nonowners. Firearm owners were significantly older than nonowners; however, this difference was small in magnitude (47.42 vs. 46.44 years). Additionally, firearm owners were significantly more likely to be male (*OR* = 2.12, 95% CI [1.93, 2.32], *p* < 0.001) and White (*OR* = 1.89, 95% CI [1.71–2.09], *p* < 0.001) compared to nonowners.

**TABLE 1:** Univariate multinomial regression analyses predicting firearm response group.

	Personally own a gun or firearm, *n* (%)			OR (95% CI)		
	No (*n* = 6380)	Yes (*n* = 2773)	Nonresponse (*n* = 1247)	Yes vs. no (ref)	Nonresponse vs. no (ref)	Nonresponse vs. yes (ref)
Age, M (SD)^a^	46.44 (17.26)	47.42 (15.51)	34.18 (14.12)	**1.06 (1.01–1.11)**	**0.43 (0.40–0.46)**	**0.41 (0.37–0.44)**
Male	2906 (45.5)	1771 (63.9)	398 (31.9)	**2.12 (1.93–2.32)**	**0.56 (0.49–0.64)**	**0.27 (0.23–0.31)**
White	3945 (61.8)	2090 (75.4)	423 (33.9)	**1.89 (1.71–2.09)**	**0.32 (0.28–0.36)**	**0.17 (0.15–0.19)**
Military service	609 (9.5)	772 (27.8)	0 (0.0)	–	–	–
Probable depression	1827 (28.6)	974 (35.1)	519 (41.6)	**1.36 (1.23–1.49)**	**1.82 (1.60–2.06)**	**1.34 (1.17–1.54)**
Probable PTSD	1283 (20.1)	864 (31.2)	399 (32.0)	**1.80 (1.63–1.99)**	**1.90 (1.66–2.17)**	1.06 (0.91–1.22)
Recent ideation (PHQ‐9)^b^	1280 (20.1)	827 (29.8)	389 (31.2)	**1.69 (1.53–1.88)**	**1.84 (1.61–2.11)**	1.09 (0.94–1.26)
Past‐month SI	234 (3.7)	169 (6.1)	81 (6.5)	**1.71 (1.39–2.09)**	**1.83 (1.41–2.37)**	1.07 (0.81–1.41)
Past‐year SI	332 (5.2)	183 (6.6)	96 (7.7)	**1.29 (1.07–1.55)**	**1.52 (1.20–1.92)**	1.18 (0.91–1.53)
SCS‐R total, M (SD)^a^	13.32 (14.32)	16.40 (18.07)	17.93 (15.08)	**1.22 (1.17–1.27)**	**1.33 (1.25–1.41)**	**1.09 (1.02–1.16)**

Abbreviations: PHQ‐9, Patient Health Questionnaire‐9; SCS‐R, Suicide Cognitions Scale‐Revised.

^a^ Values for mean and SD are raw values. Variable was standardized in logistic regression analysis.

^b^ Binary response; recent ideation was defined as a nonzero response on the ninth item of the PHQ‐9. Odds ratios in bold are statistically significant at *p* < 0.05.

Firearm owners and nonresponders were significantly more likely to endorse probable depression, probable PTSD, recent ideation, past‐month SI, past‐year SI, and more suicide cognitions (SCS‐R total score) than nonowners. Compared to firearm owners, nonresponders were significantly more likely to endorse probable depression (*OR* = 1.34, 95% CI [1.17–1.54], *p* < 0.001) and higher scores on the SCS‐R (*M* = 17.93 vs. 16.40).

### Multivariate analyses

We then examined the four aspects of suicide risk as predictors of firearm response group when accounting for demographics, probable depression, and probable PTSD (Table 2). Several commonalities and differences emerged across the four multivariate models when covariates were examined. Age, gender, race, and probable PTSD were significant predictors of firearm response group across all four models. Specifically, individuals who were male (*AOR*s = 1.93–2.00), White (*AOR* = 1.82), and endorsed probable PTSD (*AOR*s = 1.72–1.78) were significantly more likely to endorse being a firearm owner than a nonowner across all models. Nonresponders were significantly more likely to be younger (*AORs* = 0.53), female (*AORs* = 0.35–0.69), and non‐White (*AORs* = 0.30–0.54) compared to firearm owners and nonowners. Nonresponders were significantly more likely to endorse probable PTSD (*AOR*s = 1.32–1.35) than nonowners but less likely to endorse such symptoms than firearm owners (*AOR*s = 0.76–0.78). Interestingly, across all four models, the effect of probable PTSD was larger in magnitude compared to the other predictors. Diverging across models, probable depression (*AOR*s = 1.14–1.16) was significantly associated with increased likelihood of being a firearm owner than a nonowner in the models examining past‐month and past‐year SI as predictors, but not in the recent ideation model.

**TABLE 2:** Multivariate multinomial regression analyses predicting firearm response group.

	Yes vs. no (ref)	Nonresponse vs. no (ref)	Nonresponse vs. yes (ref)
	AOR (95% CI)	AOR (95% CI)	AOR (95% CI)
Recent ideation model			
Age^a^	1.01 (0.96–1.07)	**0.53 (0.49–0.58)**	**0.53 (0.48–0.58)**
Male	**1.93 (1.75–2.12)**	**0.69 (0.60–0.79)**	**0.36 (0.31–0.42)**
White	**1.82 (1.63–2.04)**	**0.54 (0.47–0.62)**	**0.30 (0.25–0.35)**
Probable depression	0.98 (0.86–1.12)	1.09 (0.92–1.29)	1.11 (0.91–1.34)
Probable PTSD	**1.72 (1.53–1.93)**	**1.34 (1.15–1.56)**	**0.78 (0.66–0.92)**
Recent ideation (PHQ‐9)^b^	**1.43 (1.24–1.64)**	1.08 (0.91–1.30)	**0.76 (0.62–0.93)**
Past‐month SI model			
Age^a^	1.00 (0.94–1.05)	**0.53 (0.49–0.58)**	**0.53 (0.49–0.58)**
Male	**2.00 (1.82–2.19)**	**0.69 (0.61–0.79)**	**0.35 (0.30–0.40)**
White	**1.82 (1.62–2.03)**	**0.54 (0.46–0.62)**	**0.30 (0.25–0.35)**
Probable depression	**1.14 (1.02–1.28)**	1.12 (0.97–1.30)	0.98 (0.83–1.16)
Probable PTSD	**1.76 (1.57–1.98)**	**1.35 (1.16–1.56)**	**0.76 (0.65–0.90)**
Past‐month SI	**1.35 (1.08–1.69)**	1.06 (0.80–1.41)	0.79 (0.58–1.07)
Past‐year SI model			
Age^a^	1.00 (0.94–1.05)	**0.53 (0.49–0.58)**	**0.53 (0.49–0.59)**
Male	**2.00 (1.82–2.20)**	**0.69 (0.61–0.79)**	**0.35 (0.30–0.40)**
White	**1.82 (1.63–2.04)**	**0.54 (0.46–0.62)**	**0.30 (0.25–0.35)**
Probable depression	**1.16 (1.04–1.30)**	1.13 (0.98–1.31)	0.97 (0.83–1.14)
Probable PTSD	**1.78 (1.58–1.99)**	**1.35 (1.16–1.57)**	**0.76 (0.64–0.90)**
Past‐year SI	1.16 (0.95–1.41)	0.96 (0.75–1.24)	0.83 (0.63–1.10)
Suicide cognitions model			
Age^a^	1.00 (0.95–1.06)	**0.53 (0.49–0.58)**	**0.53 (0.49–0.58)**
Male	**1.97 (1.79–2.17)**	**0.69 (0.60–0.79)**	**0.35 (0.30–0.41)**
White	**1.82 (1.63–2.03)**	**0.54 (0.46–0.62)**	**0.30 (0.25–0.35)**
Probable depression	1.09 (0.96–1.24)	1.07 (0.90–1.26)	0.98 (0.81–1.18)
Probable PTSD	**1.73 (1.53–1.95)**	**1.32 (1.13–1.54)**	**0.77 (0.65–0.91)**
SCS‐R Total^a^	**1.07 (1.01–1.14)**	1.06 (0.97–1.15)	0.99 (0.90–1.08)

Abbreviations: PHQ‐9, Patient Health Questionnaire‐9; SCS‐R, Suicide Cognitions Scale‐Revised.

^a^ Variable was standardized in analysis.

^b^ Binary response; recent ideation was defined as a nonzero response on the ninth item of the PHQ‐9. Odds ratios in bold are statistically significant at *p* < 0.05.

Associations between the suicide‐related variables of interest and ownership group were somewhat consistent across models. Firearm owners were significantly more likely to endorse recent ideation (*AOR* = 1.43, 95% CI [1.24–1.64]; *p* < 0.001), past‐month SI (*AOR* = 1.35, 95% CI [1.08–1.69]; *p* = 0.008), and higher scores on the SCS‐R (*AOR* = 1.07, 95% CI [1.01–1.14]; *p* = 0.026) than nonowners. Contrasting with these findings, however, past‐year SI was not associated with ownership. Moreover, nonresponders were significantly less likely to endorse recent ideation than firearm owners (*AOR* = 0.76, 95% CI [0.62–0.93]; *p* = 0.007) in the recent ideation model, but nonresponse was otherwise not associated with suicide ideation or suicidal cognitions in the remaining models.

## DISCUSSION

Firearm ownership and access is associated with increased suicide risk. Yet, many individuals do not respond to questions about firearm ownership in research studies, hampering efforts to characterize these relationships, and ultimately, inform intervention and prevention efforts. To build upon recent findings from Bryan et al. (2021), this study examined whether key demographic, psychiatric, and suicide risk‐related factors differentiated individuals who endorsed or denied firearm ownership from nonresponders. At the univariate level, we unexpectedly found that nonresponders tended to be younger, were more likely to be female, and less likely to be White than responders (i.e., firearm owners and nonowners). Both nonresponders and firearm owners had higher rates of probable depression and PTSD, suicide ideation across multiple time frames, and stronger suicide‐related cognitions. When accounting for key demographic and psychiatric covariates in multivariate models, nonresponders and responders showed largely similar levels of suicide risk across measures. Overall, the current findings have important implications informing the assessment of firearm ownership and firearm suicide prevention efforts.

Unexpectedly, we found that individuals who provided a nonresponse to an item assessing firearm ownership were not demographically similar to respondents, regardless of firearm ownership status. Instead, our results suggest that nonresponders are more likely to be younger, female, and non‐White, indicating distinct differences from both respondent groups. There is some evidence that individuals who are female and/or from underrepresented racial groups may be less comfortable to share their firearm ownership status due to social desirability effects (Legault, 2013; Ludwig et al., 1998), especially if they deviate from their typical group norms. Moreover, individuals who perceive themselves as stigmatized or who may be worried about being stigmatized may be more reluctant to self‐identify as owning firearms (Kleck et al., 2009), and these concerns may be compounded in the context of recent events in the US (i.e., Black Lives Matter; police killings of Black individuals). Data for the current study was collected from March to April 2020, which was during the beginning of the COVID‐19 pandemic in the US and a racial justice movement in response to police brutality toward Black, Indigenous, and other racial and ethnically underrepresented communities. During this time, there was a surge in firearm purchases, particularly among first‐time purchasers (Curcuruto, 2020) and individuals who do not fit the “typical” depiction of firearm ownership in the US (i.e., White, older, and male). It is estimated that among new firearm owners in 2020, almost 50% were female, 21% were Black, and 19% were Hispanic (Miller et al., 2022). Interestingly, the demographic shifts in new firearm ownership started in 2019, with first‐time firearm owners in 2019 and 2020 being less likely to be White, male, and were more likely to be younger than other firearm owners (Miller et al., 2022). Thus, the current study's findings may possibly reflect recent shifts in the demographic characteristics of firearm owners.

Nevertheless, it is worth noting that nonresponse may not necessarily indicate ownership status. It may be that individuals are hesitant to disclose their firearm ownership status due to fears of what others may think. For instance, Wallace (2017) found that 60% of individuals who expressed fear of what others may think were nonowners, and that nonowners reported less comfort sharing their firearm ownership status (Wallace, 2017). Additionally, White, male, and older individuals with stronger progun attitudes—which represents similar demographic characteristics of the majority of firearm owners in the US (Schaeffer, 2023)—reported more comfort in disclosing ownership status, regardless of being an owner or nonowner (Wallace, 2017), which may explain why these demographic characteristics differentiated responders from nonresponders in the current study. It may be that individuals who do not fit the “typical” demographics of a firearm owner in the US are more reluctant to disclose their firearm ownership status. It is therefore likely that the nonresponders group represents a heterogenous mix of individuals who are nonowners and firearm owners. This may explain the observed patterns of differences, and lack thereof, between nonresponders and the other two groups. Specifically, nonresponders did not significantly differ from either responding group on probable depression, were more likely to endorse probable PTSD than nonowners, and were less likely to endorse probable PTSD and recent SI on the PHQ‐9 than firearm owners.

Importantly, findings suggest that individuals who endorse firearm ownership may be at elevated risk for suicide, especially compared to nonowners. In multivariate models, firearm owners endorsed higher rates of past‐month suicidal ideation, and higher suicide‐related cognitions than nonowners, and higher rates of recent ideation than nonowners and nonresponders. This is especially concerning given that these individuals are at heightened risk for suicide and have personal access to a firearm, a highly lethal mean for suicide. Our finding suggests that personal firearm ownership in particular may be associated with elevated rates of suicide ideation, which differs from prior research indicating that households with and without firearm access have similar rates of suicide ideation (Betz et al., 2011; Ilgen et al., 2008; Miller et al., 2009). The prior studies were conducted more than a decade ago, and the current findings may reflect recent shifts in the demographic characteristics of firearm owners (e.g., women, individuals from underrepresented ethnic and racial backgrounds). For instance, firearm ownership rates have increased among women (Brenan, 2022), who tend to have higher rates of suicidal thoughts than men (Ivey‐Stephenson et al., 2022). However, further research examining if these patterns can be replicated is needed before such conclusions can be determined. Despite this, the findings underscore the importance of implementing suicide prevention strategies with firearm owners. Brief interventions, such as lethal means counseling (LMC), may be beneficial. In particular, a motivational‐interviewing‐based LMC approach that helps individuals identify their reasons for and against changing their access to firearms may foster collaboration and engagement (Britton et al., 2016; Bryan et al., 2011). Such an approach has been found to be effective in promoting secure firearm storage practices among National Guard personnel (Anestis et al., 2021) and would be especially beneficial as a primary prevention approach since 90% of suicide attempts with a firearm result in death (Spicer & Miller, 2000).

We also observed that firearm owners were more likely to endorse probable PTSD than nonowners and nonresponders, and nonresponders were more likely to endorse PTSD than nonowners. Trauma exposure and PTSD are established risk factors for suicidal thoughts and behaviors (e.g., Nock et al., 2009), and the rate of suicide deaths may be particularly elevated among women with PTSD (Fox et al., 2021). It may be that firearm owners and nonresponders have elevated risk factors for suicide than nonowners. Specifically, individuals who endorsed firearm ownership in the current sample may be at a particularly elevated risk for suicide given their recent experiences of suicide‐related thoughts and cognitions and PTSD symptoms. Those in the nonresponse group may also be at elevated risk for suicide, albeit lower than firearm owners, due to nonresponders being more likely to endorse probable PTSD and to be female. Our findings are consistent with some studies implicating current PTSD in firearm ownership (Stanley et al., 2020), and it may be that individuals who have lingering effects from trauma are more prone to own a firearm to increase perceptions of safety, and are more comfortable acknowledging personal firearm ownership.

It is also worth noting that this study was designed to be a replication and extension of Bryan et al.'s (2021) research. The current study's findings diverged from Bryan et al. (2021), who found similarities in psychiatric distress and suicide risk across those with firearm access and those who selected “refuse to answer”. Key differences in study design may, in part, explain the divergent findings. First, the sample in the study by Bryan et al. (2021) consisted of current or retired US military service members presenting at a military primary care clinic, while the current study's sample consisted of community members recruited nationally to participate in an anonymous online survey. Specifically, participants' responses in the military sample were identifiable, and research suggests that military personnel may underreport mental health symptoms and suicidal ideation on identifiable questionnaires compared to anonymous questionnaires (Vannoy et al., 2017). Yet, it is important to consider that the current study's sample was recruited from a larger pool of individuals who had expressed interest in completing online surveys and may not be representative of the US population. Second, Bryan et al. (2021) used an item assessing household firearm access (“Are any firearms now kept in our around your home?”), while the current study assessed personal firearm ownership (“Do you personally own a gun or firearm?”). Finally, we examined those who did not provide an answer to the item while Bryan et al. (2021) explored those who selected the response option of “refuse to answer”. Thus, the different patterns of results may partially reflect that how we ask about firearm ownership and access, in what contexts, and to whom matter.

Collectively, the current findings suggest that nonresponders have elevated suicide risk. Among nonresponders, those who are indeed firearm owners but are unwilling to disclose owning a highly lethal mean may be at a higher risk for suicide and differ from the “typical” firearm owner. It is therefore important to implement strategies limiting access to firearms and other lethal means for suicide with everyone, not just those who voluntarily disclose firearm ownership or access. Given that individuals may be reluctant to disclose their firearm ownership status, suicide prevention strategies may want to incorporate techniques that community members can learn and/or implement on their own to maintain their autonomy and minimize their concerns about privacy. For instance, this may involve tailoring public health messaging to different demographic subgroups of individuals with firearm access promoting secure firearm storage practices, and in which situations individuals may want to consider changing their firearm access and the available options (e.g., temporarily storing firearm(s) outside of the home when experiencing significant stressors). However, additional research is needed to ascertain the usefulness of such a public health approach, particularly when it is tailored to specific demographic subgroups. The context of how and when firearm ownership is assessed may also be important. For example, a study examining a sample of suicide decedents who received ambulatory care from Kaiser Permanente Washington found that the majority of firearm suicide decedents who received a question about their firearm access were willing to answer it (Richards et al., 2022). An individual's perceived acceptability of being asked about firearm access may also be influenced on the rationale for asking such a question (Khazanov et al., 2022). Thus, further investigations understanding the reasons behind nonresponse to an item assessing firearm ownership in different contexts (e.g., self‐report versus interview, during an appointment with a medical or behavioral health provider, anonymous research survey) are needed. Such research could inform the development of better assessment methods for determining firearm ownership, particularly among certain subgroups, and ultimately inform alternative ways that we can reach these individuals who are reluctant to answer such an item.

This study has several limitations. First, due to small cell sizes, we were unable to consider whether individuals who selected the “refuse to answer” response about firearm ownership meaningfully differed from those who did not select a response option. Second, low rates of endorsement precluded examining response patterns among individuals who self‐identified outside of the gender binary, a group particularly warranting attention due to their elevated risk for suicide. Future research is needed to specifically focus on response patterns among those who are traditionally underrepresented as firearm owners. Third, though previous literature suggests that firearm owners are reticent to answer items about ownership, we were unable to ascertain if nonresponders to the firearm ownership were truly firearm owners. Fourth, we were unable to examine differences in firearm response groups based on military status due to 100% of nonresponders denying current or prior military service. Examining differences based on military status is an important next step that could inform if different assessment approaches may be needed for certain subgroups that are at higher risk for suicide.

## CONCLUSIONS

Despite these limitations, the present study offers an important and novel contribution to the literature on response patterns associated with firearm ownership. Findings suggest that individuals who do not respond to a firearm ownership item may be demographically different to those willing to provide an answer, regardless of their firearm ownership status. Nonresponders may reflect a heterogenous group of firearm owners and nonowners, and further research is needed to explore their unique perspectives to minimize future nonresponses in clinical and research settings.

## CONFLICT OF INTEREST STATEMENT

None.

## Data Availability

Upon request from the corresponding author.

## Endnote

^i^
Out of the 10,400 participants included in the analyses, a total of 48 (0.5%) participants reported their gender as “transgender” (*n* = 30) or “other” (*n* = 18). Due to the low responses, we treated these responses as missing values in the regression model for analytic purposes.
